# A Narrative Review of Parameters Influencing Preeclampsia in the COVID-19 Era

**DOI:** 10.7759/cureus.45479

**Published:** 2023-09-18

**Authors:** Nishi M Modi, Hafza Afrah, Odeth Baldeon Chavez, Marjorie D Barboza Rojas, Boney J Lapsiwala, Yasmin Ahmadi, Sania J Moonnumackel, Arun Nair

**Affiliations:** 1 Medicine, Government Medical College, Surat, IND; 2 School of Medicine, China Medical University, Shenyang, CHN; 3 School of Medicine, University of San Martin de Porres, Lima, PER; 4 School of Medicine, Royal College of Surgeons in Ireland - Medical University of Bahrain, Muharraq, BHR; 5 Internal Medicine, Medical City Fort Worth, Fort Worth, USA; 6 Pediatrics, Saint Peter’s University Hospital, Somerset, USA

**Keywords:** preeclampsia, posterior reversible encephalopathy syndrome (pres), neurofilament, pre-eclampsia, covid-19 in pregnancy, covid-19

## Abstract

The COVID-19 outbreak has emerged as one of the most profound medical events of the 21st century, leaving an indelible impact on a global scale. The widespread prevalence causing significant illness and death needs collaborative and inventive efforts to deal with this challenge. One of the particular subset of the general population that had endured a significant impact was the pregnant population. A key complication of pregnancy seen in patients with a COVID-19 infection was the increased risk of developing preeclampsia. The angiotensin-converting enzyme 2 (ACE2) receptor is an important part of the renin-angiotensin system, which has been implicated in the control of blood flow and also is a key receptor in the pathogenesis of the multitude of symptoms of COVID-19. This study aimed to evaluate the psychiatric, hematological, neurological, and social factors influenced by the COVID-19 virus and its subsequent effect on the development of preeclampsia. Increased rates of anxiety and depression were seen globally during the COVID-19 pandemic and due to the following physiological response of anxiety and depression, elevated blood pressure levels and development of preeclampsia were noted. Neurological factors such as the development of posterior reversible encephalopathy syndrome and its relationship between COVID-19 and preeclampsia were also strongly observed. The observation suggested biomarkers such as serum neurofilament light may be used as a screening tool to stratify the severity of preeclampsia. Hematological parameters observed were most notable for the presence of thrombocytopenia, which itself is a marker of the severity of preeclampsia. The numerous effects of COVID-19 on preeclampsia have proven to have a tremendous impact on the healthcare burden. Careful analysis and prevention strategies, if implemented, will contribute to reducing the morbidity and mortality of patients with preeclampsia and COVID-19 infections.

## Introduction and background

In December 2019, the World Health Organization (WHO) declared the novel coronavirus (nCoV) known as the severe acute respiratory syndrome coronavirus 2 (SARS-CoV-2) as the leading cause of the COVID-19 outbreak. The coronavirus is categorized as an enveloped, positive single-stranded RNA virus, and it contains four structural proteins: spike (S), envelope (E), membrane (M), and nucleocapsid (N) [1]. These proteins share a high degree of sequence similarity with the sequence of the corresponding proteins of severe acute respiratory syndrome coronavirus (SARS-CoV) and Middle East respiratory syndrome-related coronavirus (MERS-CoV). The prospects of treating this virus have been made feasible due to the large degree of similarity in the shared mechanism of pathogenesis.

SARS-CoV-2 infection during pregnancy may contribute to the pathophysiology of preeclampsia through a number of routes. The virus’s spike protein’s N-terminal region attachment to the angiotensin-converting enzyme 2 (ACE2) receptor on the cell membrane enables the entry of the virus into the cell [2]. The ACE2 receptor is a fundamental feature of the renin-angiotensin system (RAS) that converts angiotensin II into angiotensin 1-7 and is involved in the regulation of trophoblast proliferation, angiogenesis [3], and blood flow. The RAS system plays a crucial role in regulating placental function by maintaining a delicate balance between its vasoconstrictive and vasodilatory pathways. This balance is responsible for controlling uteroplacental blood flow to a great degree. Angiotensin 1-7 levels are decreased [4] as a result of SARS-CoV-2 binding to ACE2 receptors, which results in a down-regulation of the RAS system and leaves angiotensin II's vasoconstrictive and proinflammatory effects unopposed [5]. These RAS changes may contribute to the pathophysiology of preeclampsia. Women with positive real-time polymerase chain reaction (RT-PCR) test results had infected syncytiotrophoblasts and inflammatory responses in the placentas. Verma et al. [6] demonstrated that SARS-CoV-2 colonizes maternal and fetal cells in the placenta that express the ACE2 receptor, which alters the regional RAS. ACE2 receptor expression was notably decreased in infected placentas. This was coupled with an increase in fms-like tyrosine kinase 1 production and a drop in proangiogenic factors. Women infected with SARS-CoV-2 had substantially higher serum levels of the preeclampsia indicators, such as sFlt-1 and angiotensin II type 1-receptor autoantibodies, compared to women who were not infected with SARS-CoV-2 before giving birth. Hence, the results of this study provide a link between SARS-CoV-2 infection and preeclampsia.

Preeclampsia is a multisystem illness that affects between 1.5% and 16.7% of pregnancies globally and between 3% and 8% of pregnancies in the US. It causes 60,000 maternal deaths and more than 500,000 premature births annually [3]. Twenty weeks after concept, preeclampsia manifests as new-onset hypertension. Preeclampsia can cause various end-organ diseases, such as proteinuria, acute kidney injury, hepatic dysfunction, hemolysis, thrombocytopenia, ruptured liver, seizures, stroke, and death [3]. Obstetric history suggesting risk factors for preeclampsia includes pregnancy weight, age, prior and family history of preeclampsia, being a primigravida, and changes in lifestyle during pregnancy. Preeclampsia develops in two stages. The first stage consists of a defective placentation in the early stages of pregnancy, whilst the second stage occurs after 20 weeks of gestation, as maternal syndrome. The excessive release of antiangiogenic substances in the blood distinguishes the maternal syndrome stage [4]. The sole treatment for this condition is placental excision and delivery of the fetus, as its true etiology remains unclear.

In this study, multiple variables were investigated to evaluate the potential correlation between COVID and preeclampsia. A thorough analysis is conducted by incorporating a wide range of indicators involving social, neurological, hematological, psychological, and family history. The impact of COVID-19 on preeclampsia was exacerbated by a multitude of psychological factors. The uncertain circumstances surrounding the pandemic gave rise to heightened anxiety, while significant lifestyle changes led to depression. Fear of infection triggered panic attacks, and disruption in circadian rhythm resulted in sleep disturbances, all of which contributed to the worsening of preeclampsia. The most common hematological manifestation caused by the virus was thrombocytopenia, which was primarily due to increased platelet activation and platelet aggregation. Ultimately, these processes resulted in an accelerated rate of platelet consumption, which led to a decreased platelet count.

Hypertension is known to cause preeclampsia, deemed to be caused by low platelet counts. Unfortunately, the social implications, such as domestic violence, increased as a result of individuals being pressured to quarantine during lockdowns. Domestic abuse incidents and hotline calls both increased after January 2020, according to police data [7]. The social dynamics of the pandemic have introduced additional challenges for pregnant individuals experiencing domestic abuse, leading to heightened levels of stress. This heightened stress, combined with the fear of obtaining the virus, contributes to their immediate well-being, and is a major psychological risk factor [8]. Neurological parameters have revealed the significant involvement of the neurofilament light chain (NFL), which serves as a biomarker that can be used to predict the occurrence of preeclampsia. Additionally, family history factors, such as diabetes, non-white ethnicity, hypertension, and cardiovascular disease, have been shown to be linked to a higher risk of progression of preeclampsia, particularly in patients affected with COVID-19.

In this literature review, we aim to examine the correlation between COVID-19 and preeclampsia with concurrent assessment of multi-systemic variables. These variables may play a role in the propagation or exacerbation of preeclampsia within the population. Through the assessment of these variables, we also aim to find methods of enhancing the management and treatment of pregnant women who contract the virus.

## Review

Psychiatric factors

The COVID-19 pandemic and the later quarantine measures have substantially impacted mental health, leading to an increase in various psychological symptoms. These symptoms include anxiety, irritability, anger, fear, sadness, guilt, panic attacks, sleep disorders, depression, and even suicidal thoughts. The combination of uncertainty, a high contagion rate, and a significant risk of mortality creates a climate of fear and stress that particularly impacts vulnerable populations, such as pregnant women. This fear arises from the potential for both the mother and the developing fetus to be affected by the virus [9].

Stress has been shown to be a potential risk factor in the occurrence of preeclampsia among pregnant individuals. This connection stems from the physiological effects that stress triggers, culminating in an elevation of blood pressure [10]. These effects manifest through the stimulation of vasoactive hormones and heightened levels of catecholamines leading to increased peripheral vascular resistance, an overactive resistance in uterine blood vessels. Stress during pregnancy can lead to a complex cascade of hormonal responses, which consists of the release of vasoactive substances, which can potentially disrupt the delicate balance of blood pressure regulation. The surge in these substances can induce constriction of blood vessels, contributing to an increase in peripheral vascular resistance. Additionally, elevated levels of catecholamines further enhance this resistance, potentially exacerbating the rise in blood pressure. Another aspect influenced by stress is the sympathetic nervous system, which becomes hyperactive in response to stressors. This heightened activity can intensify the constriction of blood vessels and contribute to an overall elevation in blood pressure. The complex interplay between stress and the sympathetic nervous system can further disrupt the delicate equilibrium necessary for maintaining healthy blood pressure during pregnancy. Furthermore, stress-induced changes in uterine vascular resistance in the blood vessels supplying the uterus can compromise blood flow to the placenta, potentially leading to inadequate oxygen and nutrient supply to the developing fetus. This impaired uterine circulation can further contribute to the rise in blood pressure observed in preeclampsia.

We have demonstrated a connection between COVID-19 infection and increased susceptibility in pregnant women to respiratory pathogens. This susceptibility arises from physiological changes, like cell-mediated immunity, potentially strengthening the link between COVID-19 and subsequent preeclampsia development [11]. COVID-19-induced major changes in lifestyle, such as home quarantine, reduced physical activity, decrease in exposure to sunlight, and increased use of electronic devices, were shown to contribute to depression [9]. We demonstrated that selective serotonin reuptake inhibitors (SSRIs) and tricyclic antidepressants (TCAs) were used to treat depression and were associated with a two- and three-fold increased risk of preeclampsia [12]. TCAs bind to a number of other receptors, including muscarinic, cholinergic, histamine H1, and α1-adrenergic receptors, which might play an etiologic role [13]. While, SSRIs increase placental chorionic vein, umbilical artery vasoconstriction, uterine artery blood flow, and fetal oxygenation were shown to be transiently decreased after fluoxetine [14].

The fear of contracting the virus can induce panic attacks, which leads to disruptions in the individual’s breathing pattern. Hyperdynamic breathing causes respiratory acidosis, which is compensated by metabolic alkalosis. Thus, alkalosis may worsen fetal oxygenation by reducing uterine blood flow [15]. Also, disruption in circadian rhythm due to COVID-19 causes many sleep disorders, such as sleep apnea. Sleep apnea causes ischemia toward the placenta, which produces toxins and leads to the clinical symptoms present in preeclampsia. These factors are responsible for intravascular inflammation, endothelial cell dysfunction, and activation of the hemostatic system [16].

Hematological factors

COVID-19 has an important multiorgan effect, and because of its physiopathology and relationship to ACE2, it causes renal illness and vascular effects, which induce hypertensive disorders of pregnancy (gestational hypertension, preeclampsia, and eclampsia), hepatic injury, and other conditions. In terms of hematological manifestations, thrombocytopenia has a prevalence of approximately 5% to 40% in patients with this particular infection. The underlying cause of thrombocytopenia in these cases is an increased consumption of platelets due to platelet activation and aggregation. Furthermore, having a low platelet count has been shown to have a correlation with extended length of hospitalization, suggesting a poor prognosis of the disease or an increased risk of mortality [17]. According to the guidelines set forth by the American College of Obstetricians and Gynecologists (ACOG), thrombocytopenia is a marker of the severity of preeclampsia, which further supports the increased risk of preeclampsia due to thrombocytopenia secondary to the COVID-19 infection [18].

Elevated neutrophil levels, observed in instances of infection and certain autoimmune disorders, contribute to tissue damage [17]. During the course of COVID-19, it has been noted that a notable elevation in the levels of infiltrating or circulatory neutrophils, particularly in the initial days of the illness, is present. Autopsy examinations have revealed neutrophil extravasation in various tissues, such as pulmonary capillaries, liver, and myocardial tissues in individuals with COVID-19. Neutrophils employ the NETosis mechanism, which is recognized as an important mediator of immunopathogenesis in COVID-19. NETosis is a programmed cell death in neutrophils, which extrudes DNA, antimicrobial proteins, and histones into the neutrophil extracellular trap (NET). This product is beneficial for avoiding pathogen invasion, but the excessive formation of it may cause a negative impact, such as tissue damage and autoimmune inflammation. Additionally, when activated, it can lead to thrombosis and hypercoagulability. SARS-CoV-2 can directly induce the release of NET and trigger the process of NETosis in healthy neutrophils [19]. This condition resembles preeclampsia, where increased neutrophil activation has been detected in the placenta and maternal circulation as a response to oxidative stress and inflammation. Furthermore, NETs have been reported in the intervillous space in women diagnosed with preeclampsia, contributing to the formation of thrombus in the microvasculature, which exacerbates placental ischemia [20].

Personal and family history

During the COVID-19 pandemic, family and personal history seemed to influence the progression and severity of SARS-CoV-2 infection and its associated complications. In pregnant patients, factors implicated in severe SARS-CoV-2 infection could also lead to a severe course of preeclampsia. Serrano et al. report that pregnant women exposed to SARS-CoV-2 in the first trimester face a higher risk of preeclampsia [21]. This risk is higher compared to pregnant women without the infection. The higher risk was primarily attributed to factors already present in the patient, such as higher body mass index, hypertension, or advanced maternal age. In contrast, factors such as diabetes, non-white ethnicity, or any pre-existing illness were found in COVID-19 patients. They were associated with several maternal complications, including death [7].

Another study conducted by Vimercati et al. shows that the infection tended to be more severe in pregnant women with comorbidities such as obesity, advanced maternal age, preeclampsia, diabetes mellitus, gestational diabetes, cardiovascular disease, and chronic hypertension [22]. These factors increased the likelihood of developing severe forms of COVID-19. Chronic hypertension, pre-existing diabetes before pregnancy, a previous history of preeclampsia, autoimmune diseases, high blood pressure, obesity, and conditions resulting in large placentas, such as multiple pregnancies, are noteworthy illnesses. These illnesses are important when evaluating personal, obstetric, and family history due to their increased likelihood of being inherited [23]. Chronic hypertension, pre-existing diabetes, previous diagnosis with preeclampsia, autoimmune diseases, high blood pressure, and obesity are key illnesses to consider for their inheritable potential. Conditions leading to large placentas, like multiple pregnancies, also carry a significant inheritance risk.

Socioeconomic factors

The COVID-19 pandemic has caused an unprecedented crisis in the 21st century. Pregnant women are part of a vulnerable group of the population that requires special care. The repercussions on healthcare systems are demonstrated by the lack of protective equipment, as well as the low numbers of ventilators and beds in the intensive care unit department. Remote health appointments have been implemented due to the increased susceptibility of healthcare professionals to SARS-CoV-2 infection [24]. The aforementioned factors may contribute to future mothers' hesitation in seeking appropriate antenatal care, which ultimately has an impact on the postpartum outcomes for both the mother and the newborn. The necessity of staying at home during the lockdown period has unfortunately resulted in increased incidences of domestic violence. After January 2020, surges in domestic violence and higher influxes of hotline calls were noted, according to police data [25]. Pregnant patients are also part of these statistics and suffer from domestic harassment, which increases stress levels, a known factor for hypertensive disorders such as preeclampsia.

According to Jamieson and Rasmussen [26], SARS-CoV-2 prevails among those who are economically and socially less advantaged. In Atlanta’s report, several factors were found to be associated with higher rates of COVID-19 in pregnancy, including lack of insurance, high neighborhood density, Hispanic ethnicity, and smaller household size. Likewise, information from New York indicates a higher frequency of COVID-19 infection among pregnant women who lived in households with greater crowding, neighborhoods with lower household incomes, buildings with low mean evaluated values, and high percentages of unemployment. Contrary to the findings in Atlanta’s report, larger household sizes were associated with increased infection rates [26]. In general, individuals facing these disadvantages are more prone to experiencing stress or depression, particularly pregnant women, who encounter heightened exposure. This stress is primarily attributed to the physiological and hormonal changes and bodily discomforts they undergo.

Neurological factors

The association between COVID-19 and preeclampsia is believed to have multi-systemic effects. The neurological manifestations and pathophysiology of the COVID-19 virus may increase the risk of developing preeclampsia. The neurological biomarker, serum neurofilament light chain (sNFL), is a protein formed during neural development. We demonstrated the correlation of this protein with the severity of diseases related to the loss of neurons [27]. sNFL has also been identified in cerebrospinal fluid (CSF) samples of patients with COVID-19 as well as preeclampsia. This finding prompts a further sign that sNFL can be used as a screening tool in patients with COVID-19 and preeclampsia, and possibly aid in stratifying risk to both the mother as well as the fetus. Thus, earlier intervention and better outcomes for both individuals could be achieved.

Another neurological pathology that has been linked to COVID-19 and preeclampsia is posterior reversible encephalopathy syndrome (PRES), with a prevalence of 1% to 4% in the general population [28]. PRES presents most commonly with headaches and visual disturbances and may have the development of seizures. A large population with preeclampsia presenting with headaches had been found to have abnormal neuroimaging, suggesting that patients with preeclampsia may need to be evaluated for PRES. COVID-19 infections have been described as having a high likelihood of the development of PRES. Coincidentally, treatment with steroids has been shown to reduce vasogenic edema caused by PRES, which is similar to one of the many presentations of COVID-19 [28]. Hence, the presence of PRES in patients with COVID-19 as well as preeclampsia is of great importance.

Discussion

The COVID-19 outbreak was determined by the WHO to be caused by a cutting-edge coronavirus (nCoV) designated as SARS-CoV-2. Pregnancy-related SARS-CoV-2 infection may influence preeclampsia pathogenesis in a number of ways. The RAS is an important regulator of placental function, controlling trophoblast proliferation, angiogenesis, and blood flow through the ACE2 receptor. ACE2 is a notable part of the RAS, involved in converting angiotensin II into angiotensin 1 to 7. This physiological system notably regulates the uteroplacental blood flow by carefully balancing its vasoconstrictive and vasodilatory pathways. These RAS alterations could have an impact on preeclampsia's pathogenesis. Numerous factors were explored in this study to determine whether there was a link between COVID-19 and preeclampsia. These risk variables include extra risk factors such as personal and family history and neurological, hematological, psychological, economic, and social parts. Factors such as the high risk of illness transmission, an increasing mortality rate, and the concern of simultaneous impact on both the mother and the fetus can all contribute to heightened stress levels [29]. Preeclampsia is associated with the occurrence of various mental health conditions, including melancholy, suicidal thoughts, anxiety, irritability, wrath, dread, sadness, guilt, and panic attacks. We demonstrated that these conditions disrupt uterine blood flow and resistance [30,31].

The most frequent hematological symptom of preeclampsia is thrombocytopenia. Therefore, a low platelet count will also cause severe preeclampsia in pregnant women. Infections and several autoimmune illnesses cause an increase in neutrophil numbers, which can damage tissue. NETosis is widely recognized as a pivotal player in the immunopathogenesis process of COVID-19 [19]. Women with preeclampsia have been reported to have NETs in the intervillous space. These NETs can promote thrombus formation in the microvasculature, exacerbating placental ischemia. Throughout this time, the patient's pre-existing conditions, such as a higher body mass index, hypertension, or advanced maternal age, were primarily responsible for the higher risk. Conversely, among individuals afflicted with COVID-19, these traits, in conjunction with diabetes, non-white ethnicity, or any pre-existing morbidity, were found to be closely correlated with severe maternal complications, including maternal mortality. The lack of protective gear, as well as an insufficient quantity of ventilators and beds in the intensive care unit department, highlight the impact on healthcare systems. The hesitance of mothers to seek adequate antenatal care ultimately impacts the outcomes of postpartum care for both the mother and the baby. Unfortunately, domestic violence has also increased due to people being forced to stay at home during the lockdown [32]. The higher prevalence of COVID-19 in pregnancy has been linked to factors such as lack of insurance, dense neighborhoods, Hispanic ethnicity, and smaller households.

In addition to cognitive-behavioral therapy and other approaches, psychotherapy plays a role in the treatment of stress during pregnancy. However, psychotherapy is excluded from this review because access may be limited, either due to provider availability or financial concerns. Researchers investigated that meditation and mindfulness, biofeedback, yoga, exercise, and expressive writing have been shown to be potential stress therapy methods [30]. Therefore, most of the research undertaken on these therapies is preliminary and prospective. More randomized controlled trials with larger sample sizes would majorly enhance the support for these therapies, particularly expressive writing. Depending on the clinical condition of the mother, expectant management may be appropriate for certain women who are 34 weeks pregnant. However, expectantly managed women remain at risk for sudden and severe disease progression, including mental status deterioration and the development of disseminated intravascular coagulation with associated hemorrhage. In such cases, emergency delivery and supportive therapy, including transfusion of packed red cells, platelets, and coagulation factors, may be required. Monitoring serial platelet counts and other laboratory investigations may be beneficial for determining the timing and method of administration. Following childbirth, most of the women demonstrate rapid clinical improvement, while the improvement of test indicators may occur at a slower pace [33]. Although anomalies in complement regulatory pathways have been described in some HELLP (hemolysis, elevated liver enzymes, and low platelet) patients, currently only anecdotal evidence is available to support the use of eculizumab [34]. The diagnosis of thrombotic thrombocytopenic purpura or hemolytic-uremic syndrome should be investigated in any woman who fails to demonstrate clinical and laboratory recovery within 48 to 72 hours postpartum or experiences a worsening of their clinical condition after giving birth.

## Conclusions

The COVID-19 pandemic has placed a large burden on healthcare systems and extensively elevated the risk of complications, especially among the pregnant population. An increased risk of preeclampsia was noted during the pandemic. Factors such as heightened anxiety, depression, stress, thrombocytopenia, and activation of the ACE2 receptors due to COVID-19 infection, resulting in malfunctioning of the RAS system, have all contributed to the development of preeclampsia. Many risk factors can increase the chance of preeclampsia. Hence, we need to study all these factors together carefully. By doing so, we can effectively identify and target modifiable risk factors through tailored screening regimens, all while prioritizing the enhancement of mental well-being support for expectant mothers. This holistic approach holds the potential to create a truly remarkable impact on the prevention and management of preeclampsia. This approach ensures the optimal health and well-being of both the mother and child.
